# Rational Design of V−ZnCo_2_O_4_ Nanowires on Nickel Foam: Achieving Superior Capacitance and Mechanical Resilience

**DOI:** 10.3390/molecules29235738

**Published:** 2024-12-05

**Authors:** Yucai Li, Shiwei Song, Meizhen Dai, Jian Wang, Yunjie Ke, Dong Zhang, Wenjun Liu, Guan Luo

**Affiliations:** School of New Energy, Shenyang Institute of Engineering, Shenyang 110136, China

**Keywords:** ZnCo_2_O_4_, element doping, specific capacitance, flexibility

## Abstract

The structural characteristics of electrode materials play a crucial role in their potential applications. Therefore, designing the material’s structure rationally is one of the most effective methods to achieve high-performance electrodes. In this study, V−ZnCo_2_O_4_ nanowires were synthesized on nickel foam using a simple hydrothermal method, and the prepared V−ZnCo_2_O_4_−2 electrode material exhibited a specific capacitance of 1621 C g^−1^. The potential applications of the prepared material were evaluated through device assembly, using V−ZnCo_2_O_4_−2 as the positive electrode and activated carbon as the negative electrode. The resulting device delivered an energy density of 127.5 Wh/kg, with a corresponding power density of 2700 W/kg. Additionally, the mechanical properties of the device were assessed, revealing that after multiple bends at different angles, the shape of the device remained well-preserved, further confirming its excellent mechanical stability.

## 1. Introduction

As global attention to sustainable development and clean energy increases, developing high-performance, environmentally friendly, and renewable energy storage devices has become an urgent task in today’s society [1,2,3]. Lithium-ion and sodium-ion batteries are widely favored for their exceptional energy density; however, their relatively low power density limits their application in many scenarios that require high power [4,5]. Meanwhile, while supercapacitors possess excellent power characteristics and can charge and discharge quickly, their relatively low energy density restricts their widespread use in high-energy-demand applications [6]. In this context, hybrid supercapacitors have emerged as an innovative solution. These devices combine the advantages of traditional batteries and double-layer capacitors, showcasing significant potential for enhancing the energy density of supercapacitors [7]. Hybrid supercapacitors can effectively increase energy density without sacrificing high power output, while also maintaining long cycle life, making them a promising choice for energy storage technology [8]. This development provides new insights for future energy storage solutions and aids in promoting the widespread application and development of renewable energy.

Nickel-cobalt-based oxides are widely recognized as key electrode materials for enhancing the performance of supercapacitors due to their unique electrochemical properties and excellent energy storage capabilities, garnering extensive attention from researchers [9,10,11]. In recent years, many research teams have conducted in-depth studies in this field to achieve higher energy storage and conversion efficiencies. For instance, Liu et al. employed a simple and effective solution method to successfully prepare ZnCo_2_O_4_ nanorods, which exhibited a specific capacity of 560 C g^−1^ at a current density of 1 A g^−1^, demonstrating its immense potential as a high-performance electrode material [12]. However, single electrode materials still suffer from poor performance and low stability [13,14,15]. To address this issue, doping strategies have played an essential role in the development of these materials. By doping nickel-cobalt-based oxides with other elements, their electronic structure and ionic conductivity can be significantly altered, thereby enhancing their electrochemical performance [16]. Doping can improve the material’s conductivity, increase the specific surface area, and optimize the pathways for ionic and electronic transport, effectively enhancing the specific capacity and rate performance of the electrodes [17]. Moreover, doping can also optimize the stability of the materials, reducing performance degradation during prolonged use. Vanadium (V), as a transition metal element, can improve catalytic performance by adjusting the electronic structure of the catalyst. Incorporating V into transition metal oxides such as ZnCo_2_O_4_ can effectively alter the strength of the metal-oxygen bonds, thereby optimizing the activation energy required for catalytic reactions and enhancing the activity [18]. Through these strategies, the performance of nickel-cobalt-based oxides in supercapacitor applications has been significantly improved, providing robust support for the future development of energy storage technologies.

In this study, V−ZnCo_2_O_4_ nanowires were synthesized on nickel foam using a simple hydrothermal method, and the prepared V−ZnCo_2_O_4_−2 electrode material exhibited a specific capacitance of 1621 C g^−1^. The potential applications of the prepared material were evaluated through device assembly, using V−ZnCo_2_O_4_−2 as the positive electrode and activated carbon as the negative electrode. The resulting device delivered an energy density of 127.5 Wh/kg, with a corresponding power density of 2700 W/kg.

## 2. Results and Discussion

**Figure 1:**
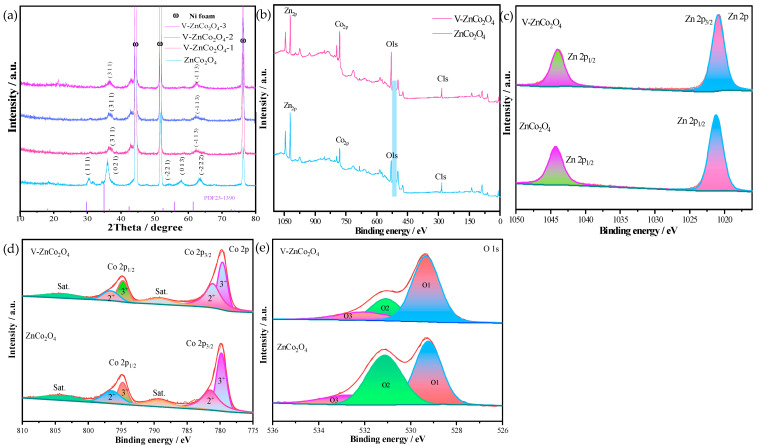
Structural features of the prepared materials. (**a**) XRD of the sample; (**b**) XPS full spectrum of the sample; (**c**) Zn 2p (**d**) Co 2p (**e**) O 1s.

Figure 1a shows the XRD patterns of different materials, the blue curve represents the XRD spectrum of ZnCo_2_O_4_, while the pink, blue, and purple curves correspond to the materials V−ZnCo_2_O_4_−1, V−ZnCo_2_O_4_−2, and V−ZnCo_2_O_4_−3, respectively. Since all materials use nickel foam as the substrate, three Ni diffraction peaks can be identified in the figure, with diffraction angles at 44.7°, 51.7°, and 77.2°. The diffraction peaks appearing at 30.5°, 35.9°, 54.0°, 58.4°, and 63.4° correspond to the (111), (021), (−211), (013), and (−222) crystal planes of ZnCo_2_O_4_ (PDF 23−1390). After doping, distinct diffraction peaks at 36.8° were observed, corresponding to the (−113) crystal plane. Figure 1b displays the full XPS spectrum of the prepared catalysts. The blue line confirms the presence of Zn, Co, O, and C elements, while the blue circles indicate the presence of V elements in the red line, confirming the successful doping of V into the ZnCo_2_O_4_ catalyst. As shown in Figure 1c, the peaks at 1044.4 eV and 1021.2 eV in the ZnCo_2_O_4_ catalyst correspond to Zn 2p_1_/_2_ and Zn 2p_3_/_2_, respectively. Compared to the V−ZnCo_2_O_4_−2 catalyst, it was observed that the V−ZnCo_2_O_4_−2 catalyst shifted by 0.3 eV towards higher energy levels, indicating that the addition of V enhanced the electronic transport rate of the catalyst and reduced the kinetic barriers during the HER and OER processes [19,20,21]. Figure 1d presents the Co 2p spectra of the V−ZnCo_2_O_4_ and ZnCo_2_O_4_ samples. The peaks at 779.7 eV and 795.1 eV correspond to Co 2p_3_/_2_ and Co 2p_1_/_2_, both of which are attributed to spin states of Co [22]. In the region of Co 2p_3_/_2_, the binding energy around 779.6 eV is associated with Co^3+^, while the binding energy near 781 eV can be identified as Co^2+^. Binding energies near 785.5 eV and 801.7 eV correspond to satellite peaks [23]. Furthermore, a comparison of the Co 2p spectra before and after V doping reveals a shift towards lower energy regions, indicating the simultaneous presence of Co^3+^ and Co^2+^ [24]. Figure 1e shows the O 1s spectra of ZnCo_2_O_4_ and V−ZnCo_2_O_4_−2, where three peaks can be observed in both materials [25]. The peak at 532.68 eV is related to low-coordinated oxygen ions, while the peak at 531.18 eV is associated with water molecules adsorbed on the surfaces and within the ZnCo_2_O_4_ and V−ZnCo_2_O_4_−2 materials [26,27]. The peak at 529.28 eV corresponds to defect oxygen.

**Figure 2:**
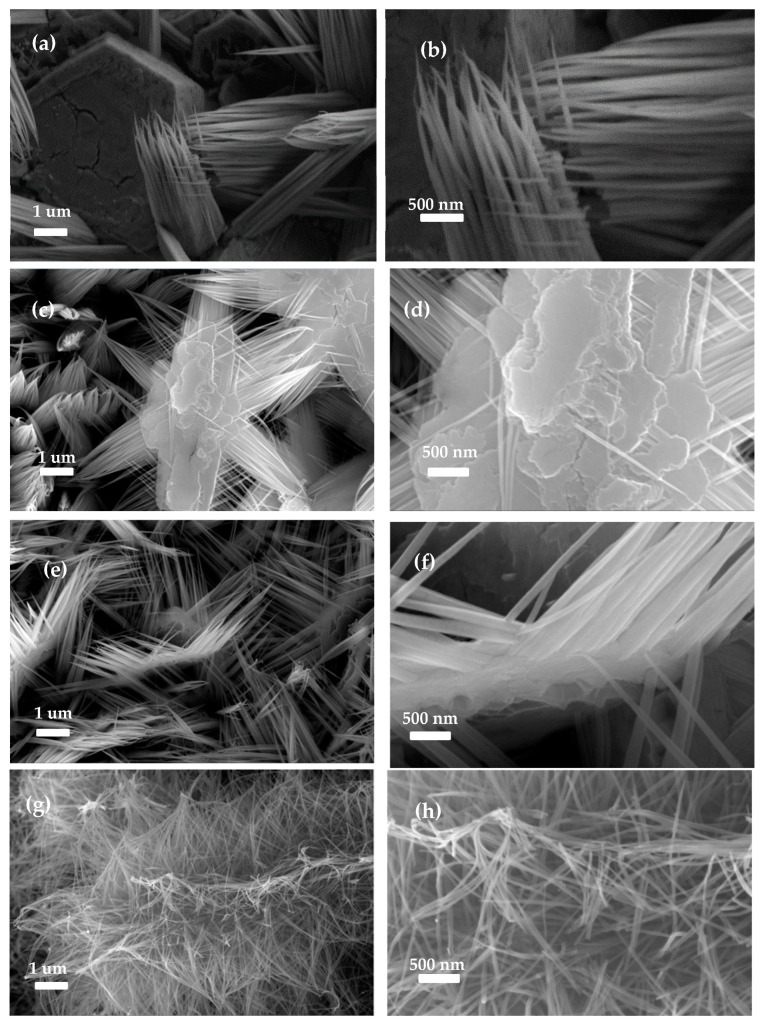
SEM images of ZnCo_2_O_4_ with different V doping levels. (**a**,**b**) are the scanning morphologies of ZnCo_2_O_4_; (**c**,**d**) are the scanning morphologies of V−ZnCo_2_O_4_−1; (**e**,**f**) are the scanning morphologies of ZnCo_2_O_4_−2; (**g**,**h**) are the scanning morphologies of ZnCo_2_O_4_−3.

The amount of V doping plays a crucial role in determining the morphology and microstructure of the produced materials. To verify the significance of V doping, comparative experiments were conducted with V−ZnCo_2_O_4_−1, V−ZnCo_2_O_4_−2, and V−ZnCo_2_O_4_−3. Figure 2a shows the low−magnification SEM image of the sample prepared with V−ZnCo_2_O_4_−1, revealing the presence of nanowires along with a small number of nanosheets. The nanosheets may have resulted from insufficient reaction time, preventing the full formation of the nanowires. Figure 2b presents the corresponding high-magnification SEM image, indicating that the average diameter of the nanowires is approximately 50 nm, with neighboring nanowires interconnected to form a network structure. The surface of individual nanowires appears rough and contains numerous pores.

As the doping amount increases to V−ZnCo_2_O_4_−2, the low-magnification SEM image in Figure 2c shows that the resulting material is evenly distributed on the nickel foam surface, forming a nanourchin structure assembled from nanowires of nearly equal diameter. The corresponding high-magnification SEM image in Figure 2d shows that the length of individual nanowires is about 3 μm, with an average diameter of 60 nm. Figure 2e illustrates the SEM image obtained at the highest doping level of V−ZnCo_2_O_4_−3, where the morphology consists predominantly of numerous nanowires. However, excessive doping leads to irregular shapes in the produced material. The high-magnification SEM image in Figure 2f indicates that the diameter of the nanowires has significantly increased, with a length of approximately 2.5 μm and an average width of 70 nm, alongside a noticeable decrease in surface roughness and porosity. This structure may hinder rapid charge transfer (Figure 2g,h). The SEM results highlight the critical influence of V doping on the material morphology, primarily due to significant variations in nucleation growth under different synthesis conditions. Therefore, selecting the appropriate V doping amount is essential for preparing electrode materials with uniform structures and unique morphologies.

**Figure 3:**
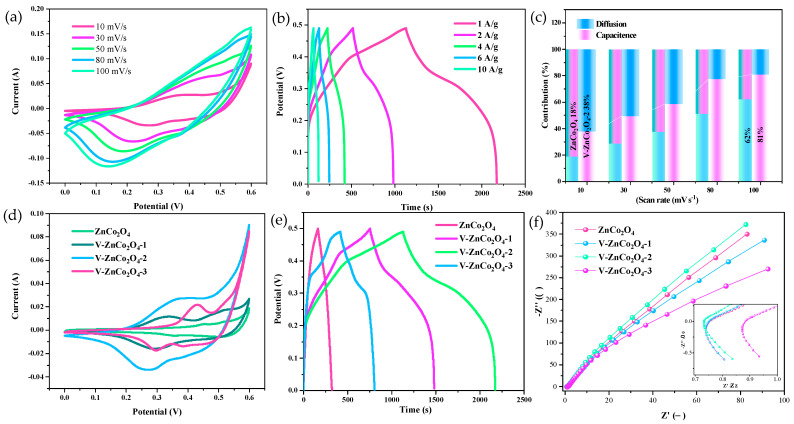
Electrochemical properties of the samples. (**a**) CV curves of V−ZnCo_2_O_4_−2 at different scanning rates; (**b**) GCD curves of V−ZnCo_2_O_4_ at different scanning scans; (**c**) Capacity Contribution Ratio; (**d**) CV curves of different samples at the same scanning rates; (**e**) GCD curves of different samples at the same current density; (**f**) Nyquist plot.

To further explore the application of the material as an electrode, we conducted an electrochemical performance study using a three-electrode system with a 3 M KOH electrolyte. Figure 3a shows the CV curves of the prepared materials at the same scan rate. It is evident that as the concentration of V elements increases, the area enclosed by the curves initially increases and then decreases. Notably, the V−ZnCo_2_O_4_−2 nanomaterial has the largest enclosed area and shows a distinct pair of redox peaks without significant polarization, indicating good rate performance [28]. Figure 3b presents the charge-discharge curves of the prepared materials at the same current density. The curves exhibit a distinct potential plateau, and their symmetry indicates excellent reversibility of the electrode. This further confirms that the prepared materials exhibit outstanding charge transfer performance, enabling faster and more stable electrochemical reactions [29]. The discharge times for ZnCo_2_O_4_, V−ZnCo_2_O_4_−1, V−ZnCo_2_O_4_−2, and V−ZnCo_2_O_4_−3 nanomaterials are 1621, 724, 1044, and 387.4 s, respectively, leading to mass specific capacities of 1621, 724, 1044, and 387.4 C/g. The results indicate that the V−ZnCo_2_O_4_−2 nanomaterial significantly outperforms the others in terms of electrochemical performance, prompting further analysis of its electrochemical characteristics. Figure 3c displays the charge-discharge curves of the V−ZnCo_2_O_4_−2 nanomaterial at different current densities, demonstrating good symmetry and stable electrochemical performance. The discharge times at current densities of 1, 2, 4, 6, and 10 A/g are 1044, 440, 200, 125, and 70 s, respectively. The mass specific capacities at these current densities are calculated to be 1044, 880, 800, 750, and 700 C/g, with a capacity retention rate of 67%, showcasing good rate performance. Figure 3d presents the CV curves of the V−ZnCo_2_O_4_−2 nanomaterial at different scan rates. The curves exhibit an increasing area with rising scan rates and clearly show the presence of redox peaks. The peak values gradually increase and shift toward both ends, but no significant polarization is observed, indicating excellent rate performance of the material. To investigate the electrochemical reaction kinetics of the V−ZnCo_2_O_4_−2 nanomaterial, we performed electrochemical impedance spectroscopy (EIS) analysis. The equivalent resistances of the ZnCo_2_O_4_, V−ZnCo_2_O_4_−1, V−ZnCo_2_O_4_−2, and V−ZnCo_2_O_4_−3 nanomaterials are 0.7508 Ω, 0.7496 Ω, 0.7326 Ω, and 0.8817 Ω, respectively. The results indicate that with increasing vanadium concentration, the equivalent resistance initially increases and then decreases. This trend is not solely due to the vanadium concentration but also reflects the complex interplay of factors such as the structural and surface properties of the material, ion diffusion, and charge transfer mechanisms. As vanadium concentration increases, the morphology and electrochemical reaction kinetics are significantly affected, leading to the observed resistance behavior. The V−ZnCo_2_O_4_−2 nanomaterial exhibits the lowest equivalent resistance, suggesting good charge transfer rates and low resistance. To comprehensively understand the electrochemical reaction kinetics of the samples, we further calculated the capacitance contribution rates of the materials, considering two energy storage mechanisms: diffusion and surface control. The capacitance contributions from both mechanisms can be quantified using the following formula [30]:

(1) $$i=k_{1}v+k_{2}v^{\frac{1}{2}}$$

where i, v, k_1_, and k_2_ represent current, scan rate, and constants, respectively. The capacitance contribution rates are shown in Figure 3f. It can be observed that the undoped ZnCo_2_O_4_ nanomaterial has a capacitance contribution of 18% at a scan rate of 10 mV/s, while the V−doped V−ZnCo_2_O_4_−2 nanomaterial has a contribution of 38%. Additionally, as the scan rate increases, the capacitance contribution of the V−ZnCo_2_O_4_−2 nanomaterial rises from 38% to 81%.

**Figure 4:**
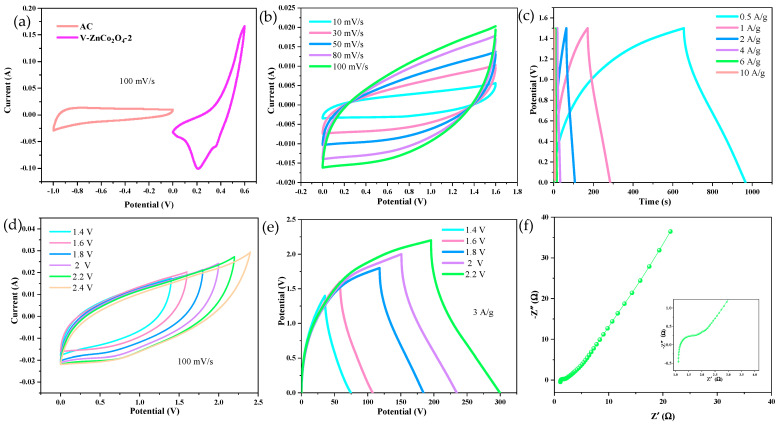
Performance of V−ZnCo_2_O_4_−2//AC asymmetric supercapacitor. (**a**) Capacitor operating voltage; (**b**) CV curves at different scanning rates; (**c**) GCD curves at different current densities; (**d**) CV curves at different voltages; (**e**) GCD curves at different voltages; (**f**) Nyquist plot.

Figure 4a shows the CV curves of the V−ZnCo_2_O_4_−2 electrode material and activated carbon at a current density of 100 mV/s. It can be observed that the voltage windows of the V−ZnCo_2_O_4_−2 electrode material and activated carbon are −1~0 V and 0~0.7 V, respectively, indicating a theoretical voltage window of 1.7 V for the device. Figure 4b presents the CV curves of the V−ZnCo_2_O_4_−2 electrode material at different scan rates. As the scan rate increases, the area of the CV curves expands, demonstrating the excellent electrochemical performance of the device. Figure 4c displays the constant current charge/discharge (GCD) curves, showcasing the charge-discharge performance of the material at different current densities. The discharge times at current densities of 0.5, 1, 2, 4, 6, and 10 A/g are 312, 112.4, 40.9, 14.5, 8.8, and 3 s, respectively. Figure 4d shows the CV curves at the same current density of 100 mV/s under different voltages. It is evident that the shape of the CV curves remains relatively consistent with increasing voltage, indicating that the device can operate stably at a voltage of 2.4 V. Figure 4e displays the GCD curves at a current density of 3 A/g under different voltages. The discharge times at voltages of 1.4, 1.6, 1.8, 2, and 2.2 V are 39, 48.7, 66, 82.9, and 104.6 s, respectively. Figure 4f presents the electrochemically measured impedance spectrum (EIS). From the inset of Figure 4f, the equivalent resistance of the V−ZnCo_2_O_4_−2 electrode material is determined to be 1.193 Ω.

**Figure 5:**
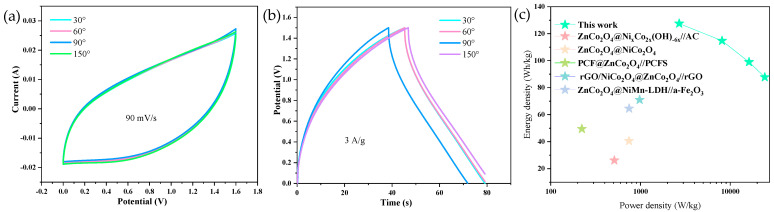
(**a**) CV curves of V−ZnCo_2_O_4_−2 at different bending angles; (**b**) GCD curves of V−ZnCo_2_O_4_−2 at different bending angles; (**c**) Energy density.

To further investigate the mechanical properties of the material, we bent the material to a certain angle and measured its electrochemical performance. As shown in Figure 5a, the material was bent to angles of 30°, 60°, 90°, and 150°, and the CV curves were measured at a scan rate of 50 mV/s. The results show that from 30° to 150°, the CV curves remain unchanged, indicating that the material possesses excellent mechanical properties and retains good electrochemical performance under significant mechanical deformation. Figure 5b shows the GCD curves of the material at different bending angles, with the position of the curves remaining largely unchanged, further confirming the mechanical performance of the material. Based on the GCD curves of the material, we calculated and plotted the Ragone plot (Figure 5c). The curves correspond to the energy density and power density calculated for different materials [31,32,33,34,35]. For the V−ZnCo_2_O_4_−2//AC system, the calculated energy densities are 127.5 Wh/kg, 114.75 Wh/kg, 99 Wh/kg, and 87.75 Wh/kg, with power densities of 2700 W/kg, 8100 W/kg, 16,200 W/kg, and 24,300 W/kg, respectively. It is evident that the asymmetric capacitor composed of V−ZnCo_2_O_4_−2//AC exhibits higher energy and power densities compared to supercapacitors made from other materials, which demonstrates the practical value of this material to a certain extent.

## 3. Experimental

In this experiment, all chemicals were used as received. Before preparing the samples, the nickel foam was subjected to surface treatment by immersing it in HCl solution, deionized water, and ethanol.

### 3.1. Preparation of V−ZnCo_2_O_4_ Electrode Material

2.5 mM cobalt nitrate, 1.5 mM zinc nitrate, 9 mM urea, and 12 mM ammonium fluoride, along with varying concentrations of V_2_O_5_ (0, 0.3 mM, 0.5 mM, 0.8 mM). These reagents were sequentially added to 40 mL of deionized water and stirred thoroughly to dissolve. The pre-treated nickel foam was immersed in this solution and heated at 120 °C for 6 h. The resulting precursor was then calcined at 350 °C for 2 h to obtain the ZnCo_2_O_4_ samples, which were named V−ZnCo_2_O_4_−1, V−ZnCo_2_O_4_−2, and V−ZnCo_2_O_4_−3 according to the V content.

### 3.2. Measurements

The structure and composition of the prepared electrode materials were verified using X-ray diffraction (Bremen, Germany). The morphology and microstructure of the synthesized samples were studied using scanning electron microscopy (SEM, Gemini SEM 300-71-31, Zeiss, Germany), and X-ray photoelectron spectroscopy (Thermo Fisher Scientific, Waltham, MA, USA) was employed to analyze the chemical bonds and surface chemical states of the materials.

### 3.3. Electrochemical Test

The electrochemical performance of the samples was investigated using a three-electrode system, with Hg/HgO as the reference electrode and Pt foil as the counter electrode in a 3 M KOH aqueous solution. The electrochemical tests included cyclic voltammetry (CV) and galvanostatic charge-discharge (GCD), conducted on a CHI660e electrochemical workstation. The mass-specific capacitance (C, C/g) of the electrode was calculated using the following equation based on the GCD curve:

(2) $$C=\frac{I\Delta t}{m}$$

where *I* represents the constant discharge current, Δt is the discharge time, and *m* is the mass of the electrode material.

## 4. Conclusions

In conclusion, this study highlights the critical role of structural characteristics in electrode materials for enhancing their performance in energy applications. The rational design of material structures is demonstrated to be an effective approach in achieving high-performance electrodes. We successfully synthesized V−ZnCo_2_O_4_ nanowires on nickel foam through a straightforward hydrothermal method, resulting in the V−ZnCo_2_O_4_−2 electrode material, which exhibited a remarkable specific capacitance of 1621 C g^−1^. The potential applications of this material were further validated through device assembly, where V−ZnCo_2_O_4_−2 served as the positive electrode paired with activated carbon as the negative electrode. The resulting device delivered an energy density of 127.5 Wh/kg, with a corresponding power density of 2700 W/kg. Moreover, mechanical assessments demonstrated that the device maintained its shape effectively after multiple bends at varying angles, underscoring its excellent mechanical stability. These findings suggest that V−ZnCo_2_O_4_ nanowires have significant potential for use in advanced energy storage systems.

## Data Availability

Data will be made available on request.

## References

[1] Zhu C.R., Wang A.L., Xiao W., Chao D.L., Zhang X., Tiep N.H., Chen S., Kang J.N., Wang X., Ding J. (2018). In situ grown epitaxial heterojunction exhibits high-performance electrocatalytic water splitting. Adv. Mater..

[2] Peurifoy S.R., Russell J.C., Sisto T.J., Yang Y., Roy X., Nuckolls C. (2018). Designing three-dimensional architectures for high-performance electron accepting pseudocapacitors. J. Am. Chem. Soc..

[3] Yan D.F., Li Y.X., Huo J., Chen R., Dai L.M., Wang S.Y. (2017). Defect chemistry of nonprecious-metal electrocatalysts for oxygen reactions. Adv. Mater..

[4] Zhao D.P., Dai M.Z., Zhao Y., Liu H.Q., Liu Y., Wu X. (2020). Improving electrocatalytic activities of FeCo_2_O_4_@FeCo_2_S_4_@PPy electrodes by surface/interface regulation. Nano Energy.

[5] Zhao D.P., Dai M.Z., Liu H.Q., Xiao L., Wu X., Xia H. (2019). Constructing high performance hybrid battery and electrocatalyst by heterostructured NiCo_2_O_4_@NiWS nanosheets. Cryst. Growth Des..

[6] Li R.Y., Xu S.L., Ai Z.Q., Qi J.G., Wu F.F., Zhao R.D., Zhao D.P. (2024). Interface engineering accelerated surface recon-struction for electrocatalytic water splitting and energy storage device through hybrid structured ZnCo_2_O_4_@NiCo-LDH nanocomposite. Int. J. Hydrogen Energy.

[7] Xu S.L., Wang S.R., Ma D.M., Li R.Y., Xiang J., Zhao R.D., Wu F.F. (2018). Improve ZnWO_4_@NiCo_2_O_4_ core-shell nanosheet arrays with regulatory interfaces and electronic redistribution. Dalton Trans..

[8] Chen C., Wang S.C., Xiong D.K., Gu M.L., Yi F.Y. (2020). Rationally designed trimetallic Prussian blue analogues on LDH/Ni foam for high performance supercapacitors. Dalton Trans..

[9] Zhao D.P., Zhang R., Dai M.Z., Liu H.Q., Jian W., Bai F.Q., Wu X. (2022). Constructing high efficient CoZn_x_Mn_2−x_O_4_ electrocatalyst by regulating the electronic structure and surface reconstruction. Small.

[10] Simon P., Gogotsi Y., Dunn B. (2014). Where do batteries end and supercapacitors begin?. Science.

[11] Xu S.L., Di Y.F., Zhao R.D., Wu F.F., Zhao D.P. (2024). Composites of NiSe_2_ nanosheets on CoMoO_4_ nanomaterials as electrodes for supercapacitors. ACS Appl. Nano Mater..

[12] Guan B., Guo D., Hu L., Zhang G., Fu T., Ren W., Li J., Li Q. (2014). Facile synthesis of ZnCo_2_O_4_ nanowire cluster arrays on Ni foam for high-performance asymmetric supercapacitors. J. Mater. Chem. A.

[13] Zhao D.P., Liu X.Y., Zhang W.C., Wu X., Cho Y.R. (2024). Highly efficient and stable Mo-CoP_3_@FeOOH electrocatalysts for alkaline seawater splitting. Small Methods.

[14] Xu S.L., Zhao R.D., Li R.Y., Li J., Xiang J., Guo F.Y., Qi J., Liu L., Wu F.F. (2024). Constructing high-performance supercapacitors and electrochemical water splitting electrode materials through core-shell structured Co_9_S_8_@Ni(OH)_2_ nanosheets. J. Mater. Chem. A.

[15] Wu Q., Jia Y., Liu Q., Mao X., Guo Q., Yan X., Zhao J., Liu F., Du A., Yao X. (2022). Ultra-dense carbon defects as highly active sites for oxygen reduction catalysis. Chem.

[16] Lin L., Ni Y., Shang L., Sun H., Zhang Q., Zhang W., Yan Z., Zhao Q., Chen J. (2022). Atomic-Level modulation-induced electron redistribution in Co coordination polymers elucidates the oxygen reduction mechanism. ACS Catal..

[17] Candler J., Elmore T., Gupta B.K., Dong L.F., Palchoudhury S., Gupta R.K. (2015). New insight into high-temperature driven morphology reliant CoMoO_4_ flexible supercapacitors. N. J. Chem..

[18] Wang J., Chang J., Wang L., Hao J. (2018). One-step and low-temperature synthesis of CoMoO_4_ nanowire arrays on Ni foam for asymmetric supercapacitors. Ionics.

[19] Li S.S., Huang L.J., Zhang Q., Lin H.J., Wang R., Feng C., Jiao Y., Chen J.R., Xu Y.C. (2024). Breaking the barriers: Engineering the crystalline amorphous interface of Fe_3_O_4_@Fe electrode material for unparalleled energy storage and water splitting efficiency. Int. J. Hydrogen Energy.

[20] Zhao Y.H., He X.Y., Chen R.R., Liu Q., Liu J.Y., Song D.L., Zhang H.S., Dong H.X., Li R.M., Zhang M.L. (2018). Hierarchical NiCo_2_S_4_@CoMoO_4_ core-shell heterostructures nanowire arrays as advanced electrodes for flexible all-solid-state asymmetric supercapacitors. Appl. Surf. Sci..

[21] Sivanantham A., Ganesan P., Shanmugam S. (2016). Hierarchical NiCo_2_S_4_ nanowire arrays supported on Ni foam: An efficient and durable bifunctional electrocatalyst for oxygen and hydrogen evolution reactions. Adv. Funct. Mater..

[22] Zhao D.P., Dai M.Z., Tong Y.L., Song X.F., Wu X. (2019). Mixed transition metal oxide nanowire arrays enabling hybrid capacitor performance enhancement. CrystEngComm.

[23] Liu H.Q., Zhao D.P., Hu P.F., Liu Y., Wu X., Xia H. (2019). Boosting energy storage and electrocatalytic performances by synergizing CoMoO_4_@MoZn_22_ core-shell structures. Chem. Eng. J..

[24] Zhao D.P., Dai M.Z., Liu H.Q., Chen K.F., Zhu X.F., Xue D.F., Wu X., Liu J.P. (2019). Sulfur-Induced interface engineering of hybrid NiCo_2_O_4_@NiMo_2_S_4_ structure for overall water splitting and flexible hybrid energy storage. Adv. Mater. Interfaces.

[25] Chen J.Z., Xu J.L., Zhou S., Zhao N., Wong C.P. (2016). Nitrogen-doped hierarchically porous carbon foam: A free-standing electrode and mechanical support for high-performance supercapacitors. Nano Energy.

[26] Dong Y.D., Xing L., Chen K.F., Wu X. (2018). Porous α-Fe_2_O_3_@C nanowire arrays as flexible supercapacitors electrode materials with excellent electrochemical performances. Nanomaterials.

[27] Sathiya M., Prakash A.S., Ramesha K., Tarascon J.-M., Shukla A.K. (2011). V_2_O_5_-Anchored carbon nanotubes for enhanced electrochemical energy storage. J. Chem. Soc..

[28] Huang L., Wei Q.L., Xu X.M., Shi C.W., Liu X., Zhou L., Mai L.Q. (2017). Methyl-functionalized MoS_2_ nanosheets with reduced lattice breathing for enhanced pseudocapacitive sodium storage. Phys. Chem. Chem..

[29] Zhao X., Cai W., Yang Y., Song X.D., Neale Z.C., Wang H.E., Sui J.H., Cao G.Z. (2018). MoSe_2_ nanosheets perpendicularly grown on graphene with Mo-C bonding for sodium-ion capacitors. Nano Energy.

[30] Shu Z.W., Shao F.Q., Bian Y.H., Liu Z.J., Shan S.P., Jiao Y., Chen J.R., Xu Y.C. (2025). Innovative strategies to Counteract Jahn-Teller effect in manganese oxide for enhanced zinc-ion battery performance. J. Power Sources.

[31] Liu T., Chai H., Jia D., Su Y., Wang T., Zhou W.Y. (2015). Rapid microwave-assisted synthesis of mesoporous NiMoO_4_ nanorod/reduced graphene oxide composites for high-performance supercapacitors. Electrochim. Acta.

[32] El-Kady M.F., Strong V., Dubin S.R., Kaner B. (2012). Laser scribing of high-performance and flexible graphene-based electrochemical capacitors. Science.

[33] Kong D.Z., Cheng C.W., Wang Y., Wong J.I., Yang Y.P., Yang H.Y. (2015). Three-dimensional Co_3_O_4_@C@Ni_3_S_2_ sandwich-structured nanoneedle arrays: Towards high-performance flexible all-solid-state asymmetric supercapacitors. J. Mater. Chem. A.

[34] Wang Q.H., Zhu L.X., Sun L.Q., Liu Y.C., Jiao L.F. (2015). Facile synthesis of hierarchical porous ZnCo_2_O_4_ microspheres for high-performance supercapacitors. J. Mater. Chem. A.

[35] Xing L., Dong Y.D., Wu X. (2018). Hierarchical Co_3_O_4_@Co_9_S_8_ nanowall structures assembled by many nanosheets for high performance asymmetric supercapacitors. RSC Adv..

